# Microenvironmental regulation of the progression of oral potentially malignant disorders towards malignancy

**DOI:** 10.18632/oncotarget.20312

**Published:** 2017-08-17

**Authors:** Ruixue Ai, Yan Tao, Yilong Hao, Lu Jiang, Hongxia Dan, Ning Ji, Xin Zeng, Yu Zhou, Qianming Chen

**Affiliations:** ^1^ State Key Laboratory of Oral Diseases, National Clinical Research Center for Oral Diseases, Department of Oral Medicine of West China Hospital of Stomatology, Sichuan University, Chengdu, Sichuan, China

**Keywords:** immunity, premalignant condition, microenvironment, oral potentially malignant disorder, therapeutics

## Abstract

Oral potentially malignant disorders (OPMD) develop in a complex tissue microenvironment where they grow sustainably, acquiring oral squamous cell carcinoma (OSCC) characteristics. The malignant tumor depends on interactions with the surrounding microenvironment to achieve loco-regional invasion and distant metastases. Unlike abnormal cells, the multiple cell types in the tissue microenvironment are relatively stable at the genomic level and, thus, become therapeutic targets with lower risk of resistance, decreasing the risk of OPMD acquiring cancer characteristics and carcinoma recurrence. However, deciding how to disrupt the OPMD and OSCC microenvironments is itself a daunting challenge, since their microenvironments present opposite capacities, resulting in diverse consequences. Furthermore, recent studies revealed that tumor-associated immune cells also participate in the process of differentiation from OPMD to OSCC, suggesting that reeducating stromal cells may be a new strategy to prevent OPMD from acquiring OSCC characteristics and to treat OSCC. In this review, we discuss the characteristics of the microenvironment of OPMD and OSCC as well as new therapeutic strategies.

## INTRODUCTION

The bidirectional communication between cells and the microenvironment is critical not only for normal tissue homeostasis [1], but also in disordered tissues. In particular, interactions among premalignant lesion cells, circumjacent stromal cells, and infiltrated immune cells represent a powerful relationship that can significantly modify the development of potentially malignant disorders [2, 3]. In 1970s, Noone and co-workers first described the relationship between the prognosis of oral squamous cell carcinoma (OSCC), a common malignancy, and T lymphocytes within the cancer microenvironment [4]. Since then, the microenvironment has been characterized in disordered tissues, including cancer and oral potentially malignant disorders (OPMD) to identify and develop novel therapies to treat OPMD and to prevent OPMD from acquiring cancer characteristics. The term “OPMD” has been used to broadly describe clinical presentations that may transform into oral carcinoma, including Oral Erythroplakia (OE), Oral Leukoplakia (OLK), Oral Lichen Planus (OLP), Oral Submucous Fibrosis (OSMF), and Actinic Keratosis [5, 6]. In the case of leukoplakia, the progress from leukoplakia to OSCC is histologically classified as mild, moderate, or severe [7]. While its progression results from heterogeneities, including aberrant mutations, there is a growing appreciation that the progression is also influenced by the microenvironment [2, 3]. As the environmental conditions keep evolving and oncogenic signals are generated during the progression from normal tissues to OPMD and subsequently to OSCC, the microenvironment also changes [8, 9]. These underscore the need to discuss the changes in the OPMD microenvironment that is dynamic. How OPMD lesional cells communicate with the microenvironment bidirectionally and promote the process in their own niche needs to be deciphered.

In this review, we summarize the current literature on the roles of different stromal cells during the progression from OPMD to OSCC and metastasis, which provides new insights on how to prevent, repress, or even reverse the progression towards malignancy. Since the microenvironment is extensive and complex and changes occur at each phase of the differentiation process from OPMD to OSCC, we chose to discuss the specific aspect of the microenvironment during the progression from OPMD to OSCC. We summarize the evidence that supports the breadth of communication within OPMD, whereby tissue cells can not only promote the progression from OPMD to OSCC, but also reverse it.

### Clinical association between inflammation, OPMD, and OSCC incidence

The etiology of OPMD is multifactorial. Tobacco and alcohol are considered major risk factors, but the role of the microenvironment is increasingly recognized as being significant in OSCC development [8, 9]. In order to visualize the abstract concept of the microenvironment, we summarized direct pieces of clinical evidence. A deregulated microenvironment affects the progress from OPMD to OSCC. More specifically, when subjected to carcinogenic factors (for example, tobacco and alcohol), the oral mucosa may induce OPMD, and lymphocyte populations may change secondarily [10]. OPMD with chronic inflammation generally supports high OSCC incidence [2, 11]. It has been reported that chronic inflammation can instigate cell proliferation and activity, which induces irreversible DNA damage [12]. Cytokines released through chronic inflammatory OLK promote carcinogenesis that, in turn, instigates the inflammatory response, forming a cyclic progression [2]. In addition, immune response failure can induce OPMD and OSCC [13–18].

### Clinical association between infection and OPMD

Oral inflammation such as human papilloma virus (HPV) infection may be associated with OPMD, indicating that a deregulated microenvironment can affect the occurrence of OPMD. For instance, HPV infection is highly prevalent in OPMD. In some studies on OPMD, HPV was detected in potentially malignant lesions [19–26]. A recent meta-analysis of 4580 specimens showed that the prevalence of HPV in normal oral mucosa, non-dysplastic leukoplakia, dysplastic leukoplakia, and in other OPMD and OSCC is 10%, 20.2%, 26.2%, and 46.5%, respectively [27]. They also detected the likelihood of HPV and showed that it is 2 to 3 times greater in OPMD than in the normal oral mucosa, while it is 4 to 5 times greater in OSCC [27]. Moreover, some reports implicated high-risk HPV genotypes, particularly HPV16 and/or 6 positive, as the most prevalent in OPMD and OSCC [28–30]. The prevalence of HPV16 and/or 6 and 18 is 33% and 14%, respectively [31]. The effect of oral *Candida* in OPMD is another well-known example [32]. For example, the prevalence of *Candida* infection in OLK, erosive OLP, and OSF is 47%, 44.29%, and 36.67%, respectively [33–35]. Other infections may be associated with OPMD such as *Helicobacter pylori* [36], HCV [37], and fungal hyphae [38], and the infection rate is about 90% [36], 23% [37], and over 25% [38], respectively.

### Clinical association between OPMD and OSCC

Chronic inflamed OPMD generally exhibits high OSCC incidence, indicating that a deregulated microenvironment can affect the occurrence of cancer [2]. For example, there is a strong association between OSCC risk and OPMD (OLK and OSMF) [11]. Although OE is less common than OLK, it exhibits a higher risk for malignant transformation; more than 90% of these red lesions show severe epithelial dysplasia or *in situ* carcinoma [39]. As for OLK, cancer occurs in 0.13% to 34% of patients per year [40]. For OSMF, the rate of malignant transformation ranges from 4% to 8% [41].

### Clinical association between OPMD with infection and OSCC

The presence of infection in OPMD may be associated with carcinomatous transformation, suggesting that a deregulated microenvironment can affect the progression of carcinomatous transformation. For instance, HPV infection in OPMD is linked to the progression of oncogenesis [28, 31, 42]. Proliferative verrucous leucoplakia, a rare and particularly aggressive form of OLK, is thought to have the strongest relationship with HPV infection [42]. This clinical disorder has a high potential for malignant transformation with a 90% malignant evolution to OSCC [43]. Regarding the effect of oral *Candida* in the development of OPMD into cancer, *Candida* infection may affect dysplasia of the epithelia and malignant transformation of OPMD [32, 44]. *Candida*-infected OLK showed a higher rate of malignant transformation on follow-up [32, 45].

### Clinical association between impaired immune responses and high OSCC incidence

Impaired immune responses can induce OPMD and OSCC. In an analysis of 122,993 patients with AIDS, the observed incidence of both AIDS-related cancers (For example, non-Hodgkin lymphoma) and non–AIDS-related cancers (For example, cancers of the tongue) were higher [13]. These results were confirmed in other studies [14–17, 46]. There is also evidence showing that adequate immune function may protect OPMD from acquiring malignant characteristics [47, 48]. In some cases of viral infections, the adequate immune system may exert its protective function either by limiting the outgrowth of virus-transformed cells or OSCC cells that require the continued expression of viral proteins for their survival, or by curbing viral replication at an earlier stage, which is called “tumorigenic hit” [47]. This contrasts with studies supporting the pro-tumorigenic functions of inflammation [49].

### Alternative therapeutic opportunities

These clinical phenomena indicate new challenges in our understanding of the functions of inflammation and suggest new treatment strategies, including reeducating immune cells in OPMD and OSCC. In such a complex environment, therapeutic effects depend on the reeducation of the stroma, which has a strong influence on the progression from OPMD to OSCC. For example, whereas macrophages can be stimulated or educated by the Th2-derived anti-inflammatory cytokines, IL-4 and IL-13, and exhibit pro-tumorigenic effects [50–52], they can also be re-educated by various pharmaceutical drugs to exhibit opposite functions [53–57]. Thus, we could take advantage of the plasticity of the OPMD stroma by re-educating cells to prevent OSCC occurrence rather than by simply ablating stromal cells.

### Progression from OPMD to OSCC; disruption of tissue homeostasis

**Figure 1:**
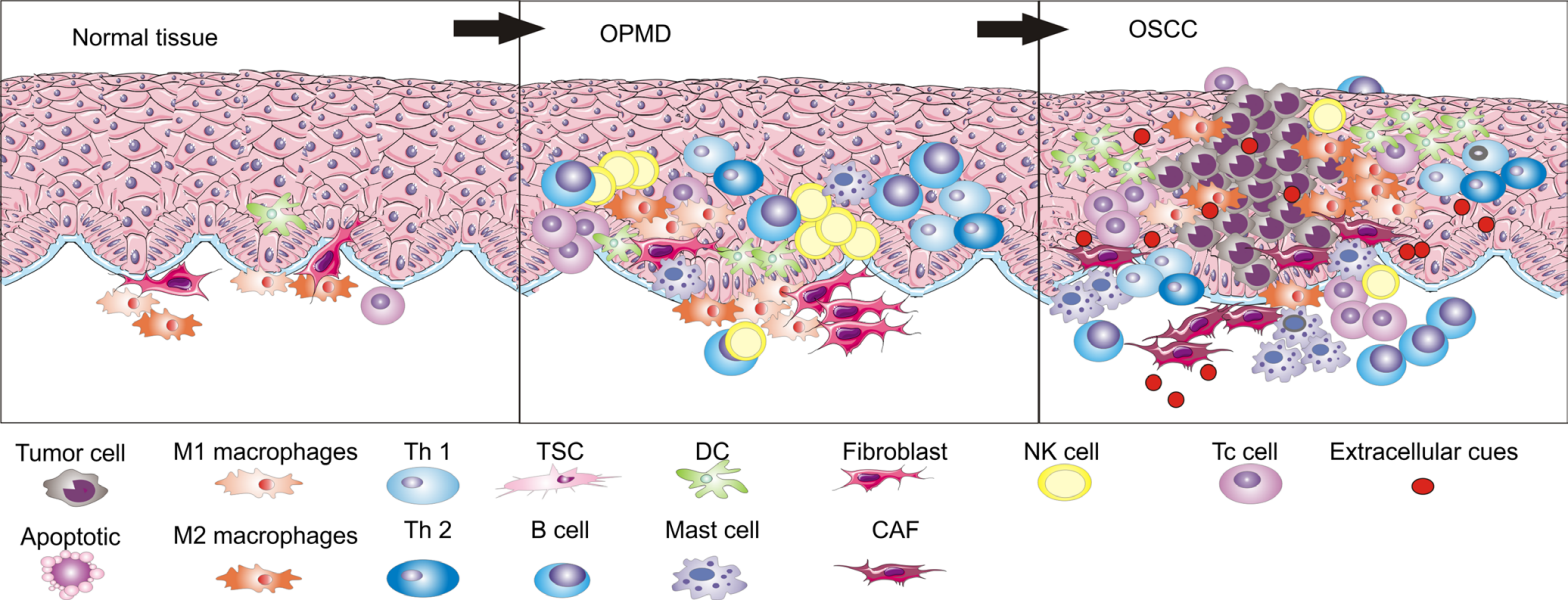
During the development process from normal epithelia to OSCC through OPMD, immune cell populations are different in number and types

**Figure 2:**
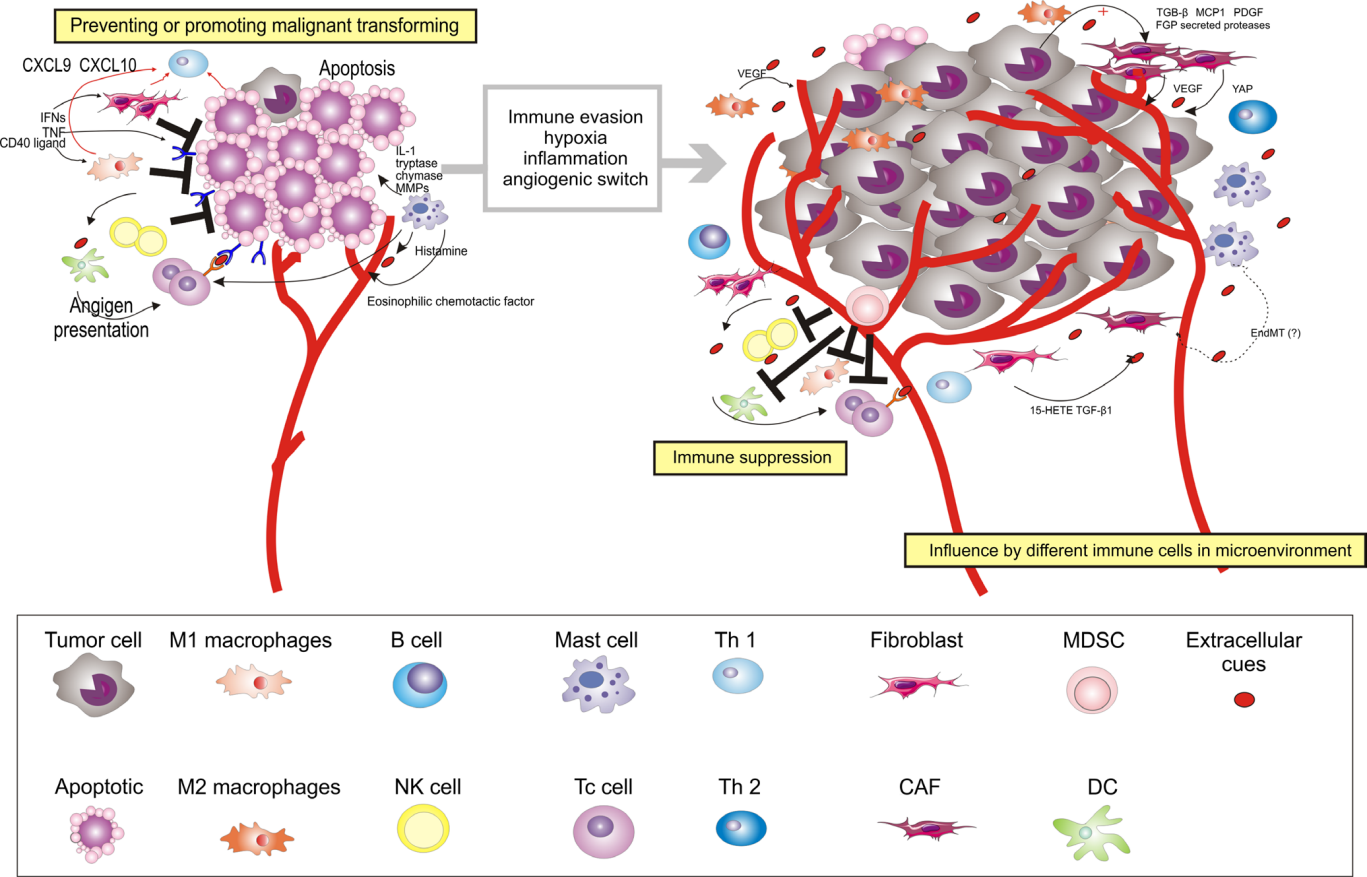
Multiple stromal cell types converge to support the development of OSCC After circumventing cell-intrinsic mechanisms of apoptosis, cancer cells are subjected to elimination pressure by the immune system. Tumor cell-specific antigens play a role during this process; they are recognized by cytotoxic immune cells, leading to their destruction. Fibroblasts, macrophages, NK cells, and Th1 cells within the microenvironment also contribute to the growth-suppressive state. Chemokines (for example, CXCL9 and CXCL10) can be induced by IFNs (IFN-gamma) and TNF or CD40 ligand, and recruit Th1 cells. After recruiting Th1 cells, IFNs (IFN-gamma) could further switch the macrophage phenotype in the oral premalignant lesions to M1 macrophages. However, fibroblasts, macrophages, and Th1 cells may later become educated by the cancer to acquire pro-tumorigenic functions. NK cells may later decrease in the microenvironment of OSCC. For instance, M2 macrophages support diverse phenotypes during the process from OPMD to OSCC, including acquisition of the malignant phenotype and angiogenesis, by stimulating IFN and secreting growth factors (for example, VEGF). As OPMD acquire a malignant phenotype, immunosuppressor cells (For example, MDSCs) are mobilized into the circulation to disrupt immune surveillance through multiple mechanisms, including, but not limited to, disruption of antigen presentation by DCs, inhibition of T_c_ cells, altering M1 macrophage polarization, and inhibition of NK cell cytotoxicity. CAFs, which become generated by cell factor (For example, TGF-β1, 15-HETE, and FSP1) activated by growth factors and cytokines (For example, TGF-β, MCP1, PDGF, and FGF), and secrete factors to support carcinogenesis (For example, VEGF). MCs aggravate the progression from OPMD to OSCC by secreting mediators, including IL-1, tryptase, chymase, MMPs, histamine, and eosinophilic chemotactic factor. In addition to cellular conditions, several extracellular properties contribute to the development of malignancy, including immune evasion, hypoxia, inflammation, and angiogenic switch. EndMT, endothelial-to-mesenchymal transition; Ag, antigen; TNF, tumor necrosis factor.

From a number of clinical associations discussed above, it is clear that the progression from OPMD to OSCC is indeed educated by an abnormal immunoenvironment. During the progression from OPMD to OSCC, the coordinated interactions between inflammatory cells that are present in epithelial tissues are disordered as the process occurs and, in turn, the tissue microenvironment regulates the carcinogenic process (Figures 1, 2, and Supplementary Table 1) [58]. We will first discuss that the stroma is hijacked for its own benefit at primary sites to escape from immune attack and to undergo the carcinogenic process to differentiate into OSCC, with a focus on the role of macrophages, myeloid-derived suppressor cells, dendritic cells, T cells, cancer-associated fibroblasts, mesenchymal stem cells, and mast cells.

### Macrophage plasticity contributes to the differentiation of OPMD into OSCC

Macrophages are essential for all animal lives. They have the ability of phagocytizing pathogens, repairing tissue, and maintaining tissue integrity [59]. They are differentiated from progenitor cells from the fetal yolk sac [60, 61], can renew by themselves [62], or are derived from the bone marrow and spleen [63]. Although they have long been regarded as important immune cells during the immune response, there is a growing appreciation that macrophages play a role in supporting multiple aspects of OPMD [59, 64]. Perhaps the most notable is that macrophages in OPMD present the M1 phenotype [64]. Indeed, a study on OLK demonstrated that the number of M1 macrophages is increased and showed a positive relationship between Th1 cells and M1 macrophages [64].

When facing different physiological conditions, macrophages can alter their polarization state functionally (Supplementary Table 1). At the extremes of their phenotypic continuum [65], macrophages can be divided into ‘Inhibit’ type macrophages (M1) and ‘Heal’ type macrophages (called M2) [59, 66, 67]. Classically activated M1 macrophages can rapidly phagocytize pathogens, inhibit cell proliferation, cause tissues damage, and act as pro-inflammatory cells [59, 67, 68], while the alternatively activated M2 macrophages can promote cell proliferation, tissue repair, tissue integrity maintenance, and act as anti-inflammatory cells [59, 67, 68]. Thus, the M1 and M2 phenotypes demonstrate the two major activities of macrophages and form a counterbalanced system. In order to be a threat to the organism from within and without, macrophages are constantly and rapidly changing their physiology [66, 69, 70]. This fluid circumstance is called macrophage plasticity [65, 66, 69, 70]. Here, we should note that, although the classification is accepted by most people and is really useful, it is somewhat too simple, as the complexity of macrophage activation has not been fully represented [65].

Currently, the functional role of macrophages in the progression from OPMD to OSCC is unclear. Environmental conditions such as the persistent type I interferons (IFN-alpha and IFN-beta)-stimulating microenvironment may mediate this transition from the premalignant to the malignant lesion [71]. By cancer immunoediting, cancer cells can acquire resistance to the antitumor responses of IFNs (IFN-alpha and IFN-beta) in the continuously stimulating environment [71]. Premalignant epithelial cells are genetically altered and are continuously stimulated by carcinogens. In this persistent stimulating environment, epithelial cells, fibroblasts, and macrophages may produce chemokines, which can recruit Th1 cells [64, 72, 73]. Some studies suggested that the expression of these chemokines can be induced by IFNs (IFN-gamma and IFN-alpha) and tumor necrosis factor (TNF) or CD40 ligand [74, 75]. These chemokines (CXCL9 and CXCL10) can be induced by IFNs (IFN-gamma and IFN-alpha), which mediate the recruitment of Th1 cells [72, 73, 76]. Indeed, it has been reported that CXCL9 is expressed in the subepithelial lesion of OLK. After recruiting Th1 cells, IFNs (IFN-gamma and IFN-alpha) could further switch the macrophage phenotype in the oral premalignant lesions to M1 macrophages [77–83]. In fact, using a mouse tumor model, a study showed that IFN-induced M1 macrophages play an important role during cancer immunoediting [84]. Furthermore, it has also been suggested that densities of M1 macrophages in tumor islets may be associated with the extended survival of patients [85, 86].

Recent evidence indicates a change to the M2 macrophage phenotype during the progression from OPMD to OSCC. A study using the WHO histopathological classification of OLK described that there was a significant increase in M2 macrophages during the development of dysplasia [64]. Other studies using pathological graded OSCC cells suggested that the number of M2 macrophages increased based on the histopathological grade [87]. Although the exact function of these cells in OLK and OSCC is unknown, evidence suggests that they may correlate with angiogenesis, OPMD malignant potential, and OSCC progression. In fact, some researchers reported that macrophage accumulation correlates with the expression of vascular endothelial growth factor (VEGF) and the density of microvessels [88, 89]. They also suggested that angiogenesis does not depend on the number of macrophages, but on their predominant phenotype [90]. Later, some studies on distinct phenotypes of macrophages showed increased infiltration of M2 polarized macrophages in early stage OSCC and OSCC with nodal lymphogenic metastasis [87, 88, 90, 91].

Taken together, these findings suggest that macrophage plasticity contributes to the progression from OPMD to OSCC. Such results support the potential of re-educating macrophages to prevent OPMD from acquiring cancer characteristics as a potential therapeutic strategy and to evaluate the prognosis of the patients.

### The interaction between dendritic cells and T cells shapes dysplastic cells

Apart from macrophage plasticity, immunosurveillance mechanisms have been described to suppress malignancy [92]. However, in an equilibrium phase, dendritic cells and T cells can interact and sculpt premalignant lesion to malignant.

**Figure 3:**
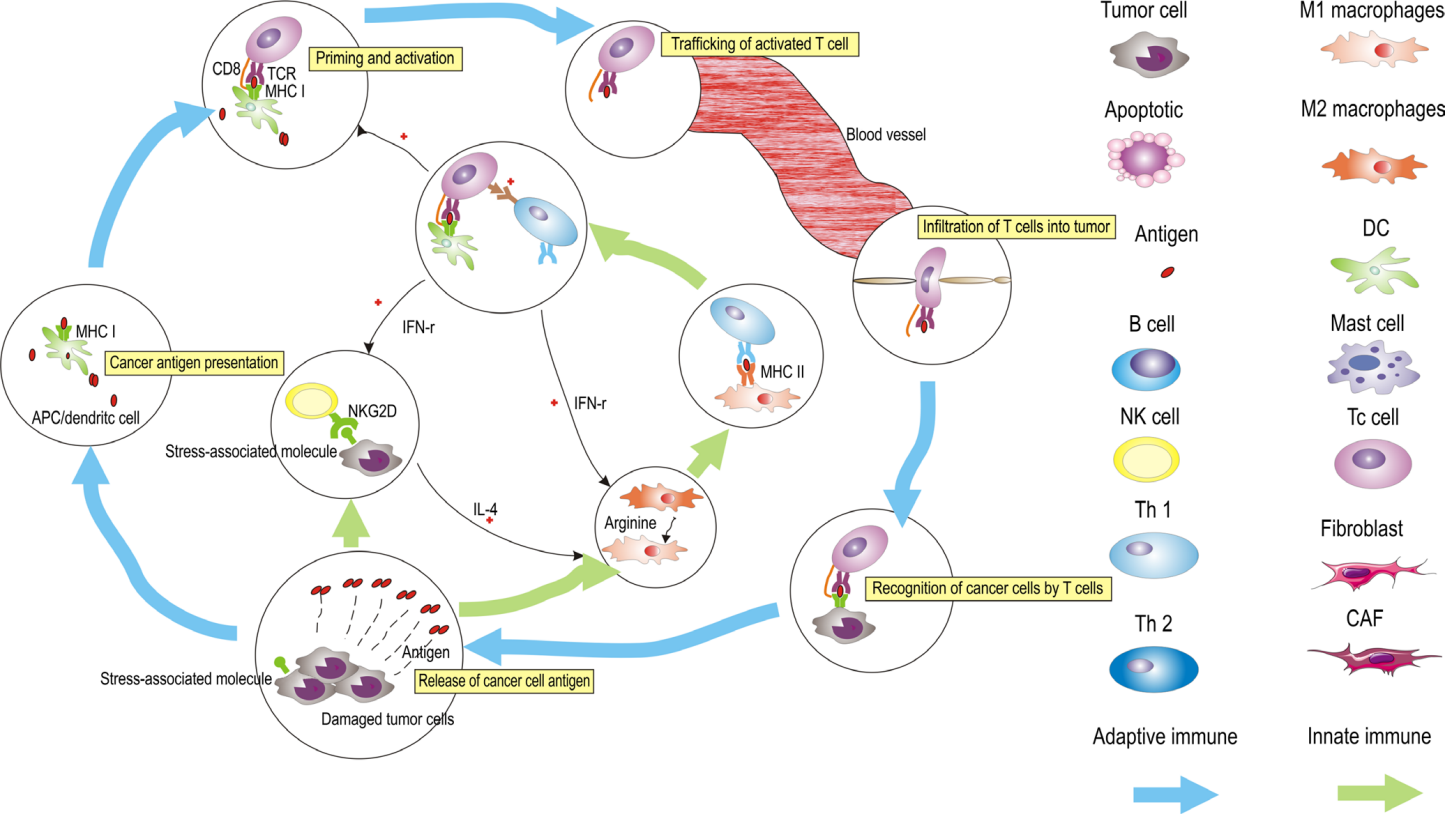
The generation of tumor immunity is a cyclic process that can be self-propagating, leading to the accumulation of immuno-stimulatory factors that, in principle, should amplify and broaden adaptive immune responses This cycle can be divided into six major steps, starting with the release of antigens from the cancer cells and ending with the killing of cancer cells, which can be regulated by innate immune cells (NK cells, macrophages, and Th1 cells). The process is described above, with the primary cell types involved. APCs, antigen-presenting cells.

Dendritic cells (DCs), potent antigen-presenting cells, can induce a T cell response [93]. DCs trap, process, and present antigens to naive or memory T cell in the context of the major histocompatibility complex class I pathway (MHC class I) [94, 95]. The presentation of antigens by DCs, which is restricted by the MHC class I, to CD8^+^ T cells generates specific cytolytic effector T cells. This is a critical step in adaptive immune response [96] (Figure 3). Dendritic Langerhans cells (LCs) is a subset of DCs present in oral mucosal linings. They can provide immunosurveillance to tissue compartments [94]. The interaction between LCs and T cells, which can select the type and direct the immune response, is crucial [94]. Given their crucial regulatory roles in directing the immune response, it is not surprising that DCs affect the progression from OPMD to OSCC. In OLK tissue, increased numbers of LCs and CD8^+^ T cells correlate with leukoplakia with dysplasia, whereas, in OSCC, the number of dendritic LCs are increased compared to that in leukoplakia with dysplasia [58]. A review indicated that the immune system, especially the interaction between DCs and T cells, and dysplasia cells or early tumor cell formation have three phases (elimination, equilibrium, and escape) [97]. In the early phase, the immune system can eliminate the damaged cells [98]. Altered self-expression by keratinocytes causes the recruitment of DCs [58]. The presence of dysplasia could result in influx of inflammation that can enhance the interplay between LCs and T cells [58]. In an equilibrium phase, by iteratively selecting and/or promoting the generation of tumor cell variants, the immune system increases the cell capacity to survive an immune attack [97, 99]. In fact, OLK with dysplasia may be an example in which proliferation of dysplastic cells and activation of the immune system are in an equilibrium phase [99]. In the escape phase, tumor, which has been sculpted by the immune system expands in an uncontrolled manner, where OPMD acquires malignant characteristics [99]. Interestingly, a recent study indicated that programmed death ligand 1 (PD-L1)-expressing dysplastic epithelial cells in OPMD may evade the host immune system [100]. Programmed death 1 [PD-1]/PD-L1 pathway, an important inhibitory checkpoint, has been highlighted recently [101]. By activating this pathway, adaptive immune resistance helps establishing an immunosuppressive microenvironment, which is one of the major mechanisms of tumor escape in the equilibrium phase [100–102]. These findings highlight potential therapeutic strategies where re-educating DCs in the equilibrium phase might prevent premalignant lesions from acquiring malignant characteristics.

### Myeloid-derived suppressor cells suppress immune surveillance

During the malignant progression from OPMD to OSCC, it is critical for cells to evade and suppress the host immune system (Figure 2), which can be achieved by inhibiting various effector immune cells or by stimulating immunosuppressive cells. It is generally agreed that myeloid-derived suppressor cells (MDSCs) are involved in immune evasion [103]. They may comprise aberrant cell populations that appear during the progression from OPMD to OSCC.

MDSCs have been functionally described as immunosuppressive, immature myeloid cells that inhibit T cell proliferative responses, antibody production, and CTL induction [104]. During tumorigenesis, MDSCs are educated, infiltrate, promote tumor vascularization, and disrupt major mechanisms of immunosurveillance by directing progenitor of DCs, activating T cells, altering M1 macrophage polarization, and inhibiting NK cell cytotoxicity [105–109]. Recent studies showed that chronic inflammation can induce MDSCs accumulation [110, 111]. When subjected to inflammation, the oral mucosa may induce OPMD, and OPMD usually gets secondarily inflamed [2, 11]. Thus, we believe that MDSCs promote the malignant progression from OPMD to OSCC. Since MDSCs include mixed subpopulations with a great diversity of plasticities and maturities and can differentiate into different types of cells, MDSCs are attractive for the design of therapies that aim at remodeling the immunosuppressive activity of MDSCs.

### Cancer-associated fibroblasts elicit pro-malignant functions in OPMD

Several important factors are involved in the malignant transformation of OPMD to OSCC such as angiogenesis, lymphangiogenesis, genetic alterations, and mutational factors [112, 113]. These changes can be achieved by activation of fibroblasts.

Fibroblasts are stromal components with diverse functions. They can deposit extracellular matrix (ECM), regulate the differentiation of connective tissue, modulate immune responses, and mediate homeostasis [114]. Cancer-associated fibroblasts (CAFs), also called peritumoral fibroblasts and myofibroblasts (MFs), are activated fibroblasts [115], similar to fibroblasts associated with wound healing [114]. In the OPMD and OSCC microenvironment, CAFs are present in abnormally high numbers that increase as the disease progresses from OPMD to OSCC and are different from normal fibroblasts [41, 116]. In general, CAFs express high levels of alpha-smooth muscle actin (a-SMA), p53, podoplanin, CD10, FAP, fibroblast specific protein 1 (FSP1), MMPs, tenascin-C, and PDGFR a/b and low levels of caveolin-1 (Cav-1) and cytokeratin [114]. In a clinical experiment, CAFs were positively detected from 0% no MFs to 21–40% MFs in OLK epithelial tissue, but not in the normal epithelium [41, 116–118]. Similarly, in OSF, the frequencies of CAFs were 100% and 86%, respectively [41, 119]. In OSCC tissue, the number of CAFs is interestingly high [41, 116, 120, 121]. As discussed above, the rate of malignant transformation of OSF is higher than that of OLK, which corresponds to the frequency of CAFs in OSF and OSCC. Another interesting study continuously observed CAF expression during the process of differentiation from normal tissue to OPMD to OSCC [116]. CAFs were not expressed in normal tissue and mild epithelial dysplasia, but expressed in severe epithelial dysplasia and OSCC [116]. These changes indicate that CAFs should be considered a cell type with potent effects on the process of differentiation from normal tissue to OPMD to OSCC, which further strengthens the “tissue organization field theory” [8, 122]. It emphasizes that mutations in epithelial/stromal cells and disturbance between stromal-epithelial interactions may be important for tumorigenesis [8, 122].

How CAFs are generated during the progression from OPMD to OSCC remains unclear. Some studies suggested that they arise from the transition of fibroblasts into myofibroblasts. Currently, the leading signal that mediates the phenotypic conversion is the transforming growth factor-β [120, 123–126]. Further *in vitro* studies suggested that 15-hydroxyeicosatetraenoic acid (15-HETE) converted fibroblasts into CAFs by the transforming growth factor (TGF)-β1-mediated FGF-2 signaling pathway [127, 128]. Such conversion is associated with the emergence of the mesenchymal marker, fibroblast-specific protein-1 (FSP1) and the down-regulation of CD31/PECAM [126]. Other studies pointed to another possible origin from endothelial-to-mesenchymal transition (EMT), vascular smooth muscle cells have different differentiation potentials to generate mesenchymal cells [120, 129–131]. Mouse experiments showed that CAFs can be generated from EMT [126]. Tumor-associated endothelial cells are not only a source for CAFs, but also promote the generation of CAFs since they can dedifferentiate to generate cells expressing CAF markers [132].

CAFs are raised and activated by growth factors and cytokines in the milieu such as TGF-β, monocyte chemotactic protein 1 (MCP1), platelet-derived growth factor (PDGF), fibroblast growth factor (FGF), and secreted proteases [120, 125].

Later, studies showed the importance of the YAP transcription factor for CAFs to remodel the microenvironment and to support carcinogenesis [133]. In turn, they also can regulate factors that re-educate the cytoskeleton and modulate matrix stiffness. This effect can induce a feedback loop to induce YAP expression.

Once CAFs are activated, they will secrete a large number of growth and proinflammatory factors. These factors play an important role in supporting carcinogenesis. For example, VEGF can permeate the vascular system and induce angiogenesis [134, 135]. Thus, the interdependency of multiple cell types may be disturbed.

### Impairment of mesenchymal stem cells is related to the progression from OPMD to OSCC

Mesenchymal stem cells (MSCs), identified in the epithelium and primarily in the basal layer [136–139], can differentiate into three germ layers and demonstrate multi-potent trans-differentiation [140, 141]. In the microenvironment of OPMD tissue, MSCs present an imbalance between regenerative and metabolic self-regulatory functions. For example, in OLK tissues, the self-renewal ability was enhanced, while the potential for trans-differentiation was decreased, compared with that of MSCs in normal tissues [142]. Indeed, during the progression of OLK, collagen IV (Col IV) was decreased and matrix metalloproteinases-9 (MMP-9) was increased in MSCs [142, 143]. Previous studies suggested that Col IV continuity was destroyed and MMP-9 positive cells were increasing in the zone adjacent to fragmented basement membranes in epithelial dysplasia and cancer [144–146]. Therefore, impairment of MSCs may be critical to destroy the basement membrane in the mucosa of OPMD and OSCC.

Why the capacity for multi-potent differentiation was greatly decreased in MSCs and concurrent with the progression from OPMD and OSCC remains unclear. The impairment of MSCs may be associated with EMT. The abnormal epithelia such as epithelial dysplasia and OSCC epithelia may interfere with EMT and produce abnormal MSCs presenting with malfunctions [142]. In addition, inflammation can reduce the multi-potent differentiation capacity of MSCs [147]. Indeed, high levels of inflammatory factors are detected in OPMD tissues [148–151].

Abnormal MSCs produced in OPMD are weaker in regulating inflammation [150]. Previous studies showed that MSCs can immunosuppress the proliferative responses of T cells via active IDO [150, 152]. However, MSCs show low immunogenicity and immunomodulatory functions in OLP [150]. In an *in vitro* study, MSCs not only immunosuppressed the proliferation of T cells directly, but also inducted T cells with a CD4+CD25+Foxp3+ regulatory phenotype [153]. All these effects contribute to the generation of an immunosuppressive environment.

### “Piece meal degranulation” of mast cells aggravates the progression from OPMD to OSCC

Mast cells (MCs), also called mastocytes and labrocytes, are large connective tissue cells, distributed preferentially in the capillaries and containing many granules rich in histamine and heparin [154]. The granules in their cytoplasm are basophilic and may obscure the nucleus [154]. In the microenvironment of oral premalignant lesions, MCs are aberrantly increased and present different phenotypes. For example, in a study comparing the number, morphology, and topographical distribution of MCs in OPMD (including OLK, OSF, and OSCC), the number of typical (TMCs), atypical (AMCs), and granular mast cells (GMCs) increased in cases with mild, moderate, and severe inflammation [155].

“Piece meal degranulation” is the characteristic trait of MCs, whereby MCs secretes certain mediators by selecting cell secretion pathways [156, 157]. The secreted mediators in OPMD play various roles in the malignant process [155]. It includes TNF-alpha, interleukin-1, heparin, histamine, chymase, bFGF, VEGF, TGF-β, IL- 8, Colony Stimulating Factor (CSF), FGF, Nerve Growth Factor (NGF), TGF, PDGF, and Stem Cell Factor (SCF) [155–159].

“Piece meal degranulation” of Mast cells aggravates the progression from OLK to OSCC. Interleukin-1, which is released by stimulated MCs, contributes to increased epithelial proliferation [160]. Histamine could facilitate the antigen to connective tissue by increasing the permeability of the mucous [160]. Similarly, studies concluded that the number of MCs increases as the differentiation from normal tissue to OLK with high-grade dysplasia and may be associated with angiogenesis [161–164]. Thus, it can be used to determine disease progression.

“Piece meal degranulation” of Mast cells aggravates the process of OSF. Similar to the number in OLK, MCs are more abundant in OSF than in the normal buccal mucosa [164, 165]. Interleukin-1, which is released by stimulated MCs, increases the fibroblastic response, which could cause the production of type-1 collagen and fibronectin. Histamine could contribute to vesicle formation and symptoms of itching sensation and submucosal edema of OSF. Eosinophilic chemotactic factor released by MCs increases the permeability of the vascellum, since eosinophils are sometimes a part of inflammatory cell infiltration [166] Archana Yadav *et al.* showed that tryptase and chymase are critical in the pathogenesis of OSF and its malignant transformation [167]. Interestingly, MCs also contribute to increased epithelial proliferation as in OLK [168].

“Piece meal degranulation” of Mast cells also aggravates the process of OLP. The number of MCs was elevated in the OLP tissues when compared with the normal tissue [164, 169]. MCs can interact with T-cells and contribute to the OLP progression in different phases of OLP [170]. It acts as a destructor of the basement membrane [170]. For example, TNF-alpha can cause the elevation of matrix metalloproteinases (collagenase), which can destruct the basement membrane and increase the expression of adhesion molecules (E-selectin and ICAM) [170]. The characteristic trafficking of lymphocytes observed in OLP is also attributed to “Piece meal degranulation” of MCs. The vasopermeable function of histamine leads to submucosal edema and proliferation of T-cells. The antigen induced T-cells cause the degeneration of basal cells, and apoptosis of keratinocytes. Thus, characteristic Civatte bodies can be detected in OLP [170].

All these studies highlight an alternative therapeutic approach by which blocking MCs’ pro-tumor mediators might prevent OPMD from acquiring cancer characteristics.

### Extracellular cues influence the progression of OPMD to OSCC

Beyond the effects of immune cells in the progression from OPMD to OSCC, the ECM has the capacity to influence the progression of OPMD. In fact, the ECM composition is a major predictor of the acquisition of a malignant behavior by OPMD. Potentially malignant oral lesions with high expression of FGF-2 and its receptors, FGFR-2 and FGFR-3, in the microenvironment are associated with the progression to OSCC [128]. OLK with high expression of tenascins and MMP-2, ECM glycoproteins, may predict the malignant potential of tobacco-associated OLK [171, 172]. Evidence also indicates that VEGFR-2 and MMP-9 protein levels are associated with epithelial dysplasia grading [173]. It has also been suggested that Nuclear factor-κB (NF-κB)-dependent cytokines (such as TNF-alpha, IL-1, IL-6, and IL-8) increase in saliva and OPMD tissues [174–177]. The NF-κB signaling pathway is critical in carcinogenesis, chemo-resistance, and protection from apoptosis in head and neck cancers [178–181]. It can mediate signal regulatory protein α (SIRPα), a cell-surface protein expressed on macrophages, to control macrophage plasticity [182]. Accumulating studies indicated that these cytokines may play critical roles in carcinogenesis and are associated with vessel density and worsened outcomes [175–177, 183]. Proteomics showed that distinct extracellular cues come from distinct cells, called the ‘matrisome’ [184]. All these data suggest that the surrounding and infiltrating extracellular cues may provide new opportunities to prevent OPMD from acquiring malignant characteristics.

### Therapeutic strategies to remodel the microenvironment

**Figure 4:**
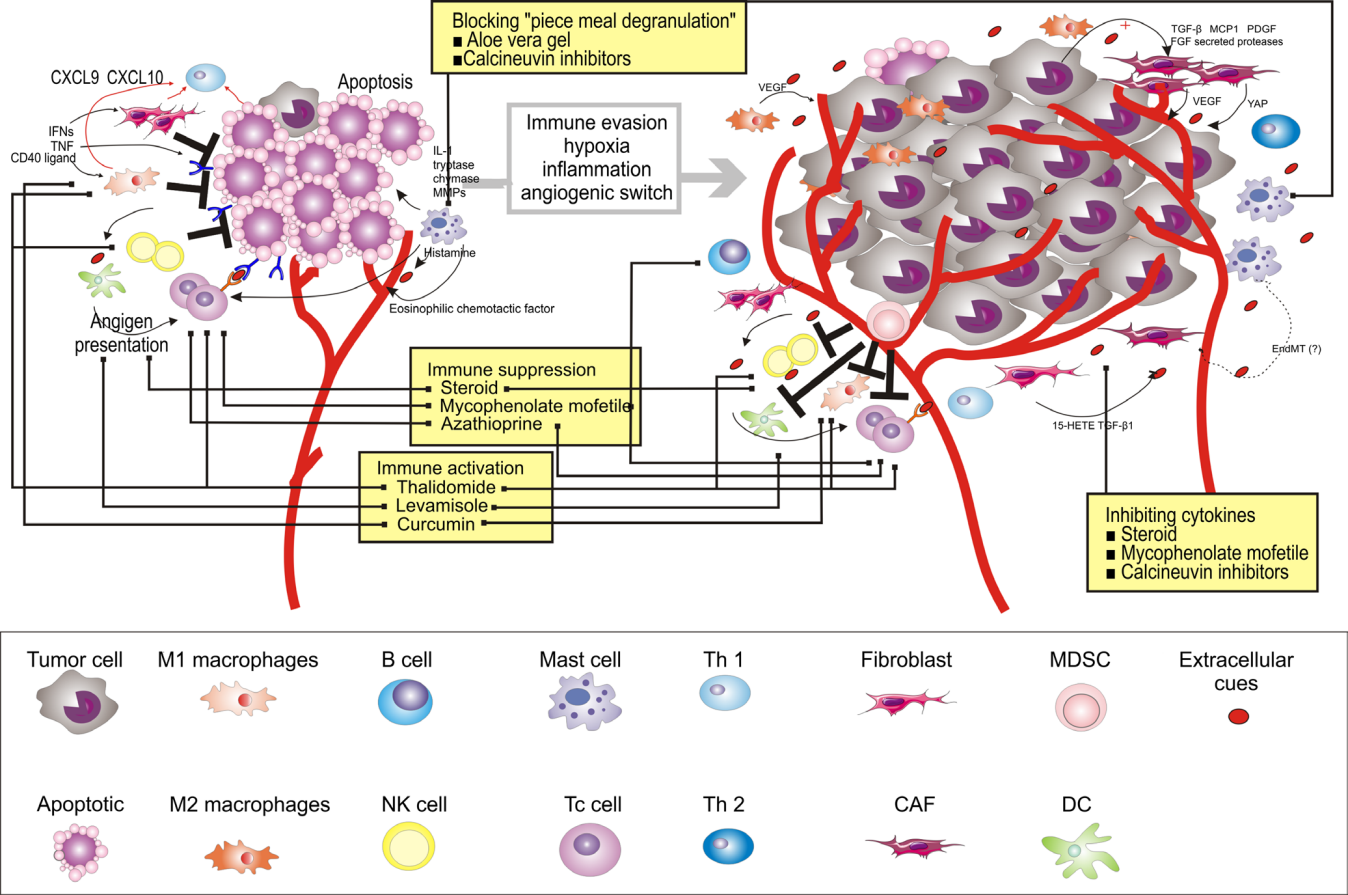
Therapeutic strategies to re-educate the OPMD (using OLP for as an example) microenvironment Multiple strategies to target the microenvironment are currently tested in clinical trials, as indicated here, and referenced throughout the review. Immune activation, marked by stimulating T cells (e.g., thalidomide and levamisole), M1 (e.g., thalidomide and curcumin), and NK cells (e.g., thalidomide), is also a promising avenue of therapeutic intervention. Sequestration of cytokines within the microenvironment, in particular T cells (e.g., steroid, mycophenolate mofetil, and Azathioprine) and B cells (e.g., steroid and mycophenolate mofetil), can be achieved by inhibiting key cytokine pathways or NF-κB signaling pathway (e.g., steroid, mycophenolate mofetil, and calcineurin inhibitors). Alternatively, “piece meal degranulation” of mast cells can be blocked by using Aloe Vera gel and calcineurin inhibitors.

Directly targeting various aspects of the cells in the OPMD tissue is the main therapeutic strategy against OPMD. However, the cells in the OPMD microenvironment are genetically stable compared to tissue lesional cells and, thus, are likely to be less susceptible to therapeutic resistance. Moreover, increasing studies indicated heterogeneity at different levels in OPMD lesional cells [185], suggesting that reeducating the microenvironment cells may become a new choice (Figure 4). Since, in the microenvironment, the cells have the paradoxical capacity of both promoting and impairing the progression from OPMD to OSCC, this plasticity must be taking into account when designing novel therapeutic strategies. One should think of manipulating and reprograming the cells in the microenvironment rather than to simply destruct or deplete the lesional cells. Although increasing immunotherapies generate exciting effects in cancer clinical trials [186–189], there is few examples of such cell manipulation and re-education approach to clinically prevent OPMD from acquiring OSCC characteristics. As the cells in the microenvironment change as OPMD progresses to OSCC, immunotherapies that can re-educate the microenvironment components may provide a new strategy to prevent OSCC.

Various therapies aimed at re-educating the immune system, many of which currently focus on patients with OLP given their high numbers of lymphocytes [190]. Thalidomide is an immune-stimulant that can inhibit TNF-alpha and co-stimulate T cells, M1, and NK cells [191]. In the clinical trial for Thalidomide in patients with OLP, significant resolution of lesion and improvement in pain was observed [192]. Several immune-stimulants have also been used. For example, levamisole, an anthelminthic drug, presents an immune-modulating effect by potentiating the activity of interleukins, interferons, and T-cell-mediated immunity [193]. Another immune-therapy success story involves a different approach, using curcumin to activate M1 [194]. Interestingly, studies demonstrated that it is effective for the management of OLP with no side effects [195]. Taken together, these studies suggest that activation of normal immune cell functionality may provide optimal benefits to the patients; however, these trials are still at an early stage with small sample sizes, and the mechanisms underlying the efficacy of these drugs remain unclear. Moreover, the evaluation of the long-term impact of these drugs on the patients’ safety will take years.

Further emerging examples of microenvironment-directed therapies that do not focus on target cell depletion include sequestration of cytokines or chemokines [196]. Among the cell types and signaling molecules discussed here, strategies that inhibit key cytokine pathways or NF-κB signaling pathway are being investigated in clinical trials [193, 195, 197–214] (Figure 4). For example, steroids, the first-line treatment for OLP, may inhibit the production of cytokines and reduce the number of immune cells [195, 197–212, 215]. Several drugs have been combined with steroids to further improve its clinical efficacy [193]. For example, Azathioprine, a synthetic purine analog, was used in combination or in sequence with a steroid to treat patients with OLP [193]. It has been reported to interfere with T-cell activation. Similarly, mycophenolate mofetil has potent cytostatic effects on B cells and T cells and, thus, inhibits chemokines [214]. In addition, calcineurin inhibitors, namely cyclosporine, tacrolimus, and pimecrolimus, exert immunosuppressive effect by inhibiting cytokine expression [213]. They can also prevent mast cells from releasing cytokines that mediate inflammation [213]. Similarly, Aloe Vera gel, a type of nutraceutical, was used in patients with OLP. The *Allianceae* family inhibits antigen antibody-mediated release of histamine and leukotriene from mast cells [216]. Four RCTs have been reported, of which three reported significantly higher overall clinical improvement [216–219]. It is plausible that microenvironment-targeted therapies should aim at manipulating and reprograming the cells in the microenvironment rather than simply destructing or depleting the lesional cells.

## CONCLUSIONS

The literature summarized in this review is encouraging for the OPMD microenvironment field and introduces new concepts and potential therapeutic strategies to reprogram the cells in the microenvironment. Nonetheless, challenges come with advances, how to identify and re-educate the cells and extracellular cues that are increasingly complex and interconnected with other components in the microenvironment remains unclear. Indeed, given that the OPMD microenvironment and mechanisms are not clear and broadly diverse during the progression from OPMD to OSCC, insights into the underlying mechanisms, how to deal with unsure response to current standard-of-care therapies by a diversity of cells will be a new challenge in this field. Another unclear question in the area is that cells in the microenvironment may be programed and sculpted by specific component(s) of OPMD and how this, in turn, results in stromal cell diversity. Additional points need be considered, including determining which patients to target, which classic therapies to combine with microenvironment-cell-targeted agents, whether intrinsic or acquired resistance like in cancer exists, and how to overcome it. However, from the studies summarized in this review, we now have a set of tools to solve these problems. For example, in order to choose specific therapies to different patients, we can select patients by analyzing the entire cells and the subset of cells in the OPMD microenvironment rather than by simply analyzing individual cells. Additionally, OPMD cell–directed agents will have to be combined with immunotherapies in a manner that allows researchers to identify the interactions between stromal cells and chemotherapies and targeted agents. Looking ahead, perhaps the most encouraging idea is that remodeling dysfunctional cells in the OPMD microenvironment could yield striking results in preventing OPMD from acquiring OSCC characteristics, as evidenced by the accumulating studies in the OPMD microenvironment field.

## SUPPLEMENTARY MATERIALS REFERENCES AND TABLES




