# Supplementary material for: Microenvironmental regulation of the progression of oral potentially malignant disorders towards malignancy
Source: Oncotarget. 2017 Aug 17;8(46):81617–35. doi: 10.18632/oncotarget.20312 (PMC5655314; doi:10.18632/oncotarget.20312)
Supplement: Supplementary file 1 [file oncotarget-08-81617-s001.pdf]

Microenvironmental regulation of the progression of oral potentially malignant disorders towards malignancy

SUPPLEMENTARY MATERIALS

Supplementary Table 1: Immune cell populations in the OPMD (using OLK for as an example) and OSCC microenvironment have distinct functions during the progression from OPMD to OSCC. See Supplementary_Table_1

REFERENCES

220. Falini B, Flenghi L, Pileri S, Gambacorta M, Bigerna B, Durkop H, Eitelbach F, Thiele J, Pacini R, Cavaliere A, Martelli M, Cardarelli N, Sabattini E, et al. PG-M1: a new monoclonal antibody directed against a fixative-resistant epitope on the macrophage-restricted form of the CD68 molecule. Am J Pathol. 1993; 142:1359–1372.
221. Lau SK, Chu PG, Weiss LM. CD163: a specific marker of macrophages in paraffin-embedded tissue samples. Am J Clin Pathol. 2004; 122:794–801.
222. Steinman RM. Decisions about dendritic cells: past, present, and future. Annu Rev Immunol. 2012; 30:1–22.
223. De Palma M, Naldini L. Tie2-expressing monocytes (TEMs): Novel targets and vehicles of anticancer therapy? Biochimica et Biophysica Acta (BBA) - Reviews on Cancer. 2009; 1796:5–10.
224. Grimm M, Feyen O, Coy JF, Hofmann H, Teriete P, Reinert S. Analysis of circulating CD14+/CD16+ monocyte-derived macrophages (MDMs) in the peripheral blood of patients with oral squamous cell carcinoma. Oral Surg Oral Med Oral Pathol Oral Radiol. 2016; 121:301–306.
225. Buim MEC, Lourenço SV, Carvalho KC, Cardim R, Pereira C, Carvalho AL, Fregnani JH, Soares FA. Downregulation of CD9 protein expression is associated with aggressive behavior of oral squamous cell carcinoma. Oral Oncol. 2010; 46:166–171.
226. Hirano C, Nagata M, Noman AA, Kitamura N, Ohnishi M, Ohyama T, Kobayashi T, Suzuki K, Yoshizawa M, Izumi N, Fujita H, Takagi R. Tetraspanin gene expression levels as potential biomarkers for malignancy of gingival squamous cell carcinoma. Int J Cancer. 2009; 124:2911–2916.
227. Ribatti D. The development of human mast cells. An historical reappraisal. Exp Cell Res. 2016; 342:210–215.
228. Ostrand-Rosenberg S. Immune surveillance: a balance between protumor and antitumor immunity. Curr Opin Genet Dev. 2008; 18:11–18.
229. Murdoch C, Muthana M, Coffelt SB, Lewis CE. The role of myeloid cells in the promotion of tumour angiogenesis. Nat Rev Cancer. 2008; 8:618–631.
230. Youn JI, Nagaraj S, Collazo M, Gabrilovich DI. Subsets of myeloid-derived suppressor cells in tumor-bearing mice. J Immunol. 2008; 181:5791–5802.
231. Lindau D, Gielen P, Kroesen M, Wesseling P, Adema GJ. The immunosuppressive tumour network: myeloid-derived suppressor cells, regulatory T cells and natural killer T cells. Immunology. 2013; 138:105–115.
232. Fridman WH, Pages F, Sautes-Fridman C, Galon J. The immune contexture in human tumours: impact on clinical outcome. Nat Rev Cancer. 2012; 12:298–306.
233. Pak AS, Wright AM, Matthews JP, Collins SL, Petruzzelli GJ, Young MR. Mechanisms of immune suppression in patients with head and neck cancer: presence of CD34(+) cells which suppress immune functions within cancers that secrete granulocyte-macrophage colony-stimulating factor. Clin Cancer Res. 1995; 1:95–103.
234. Vasquez-Dunddel D, Pan F, Zeng Q, Gorbounov M, Albesiano E, Fu J, Blosser RL, Tam AJ, Bruno T, Zhang H, Pardoll D, Kim Y. STAT3 regulates arginase-I in myeloid-derived suppressor cells from cancer patients. J Clin Invest. 2013; 123:1580–1589.
235. Gabrilovich DI, Nagaraj S. Myeloid-derived suppressor cells as regulators of the immune system. Nat Rev Immunol. 2009; 9:162–174.
236. Munera V, Popovic PJ, Bryk J, Pribis J, Caba D, Matta BM, Zenati M, Ochoa JB. Stat 6-dependent induction of myeloid derived suppressor cells after physical injury regulates nitric oxide response to endotoxin. Ann Surg. 2010; 251:120–126.

237. Lechner MG, Liebertz DJ, Epstein AL. Characterization of Cytokine-Induced Myeloid-Derived Suppressor Cells from Normal Human Peripheral Blood Mononuclear Cells. J Immunol. 2010; 185:2273–2284.
238. Lechner MG, Megiel C, Russell SM, Bingham B, Arger N, Woo T, Epstein AL. Functional characterization of human Cd33+ and Cd11b+ myeloid-derived suppressor cell subsets induced from peripheral blood mononuclear cells co-cultured with a diverse set of human tumor cell lines. J Transl Med. 2011; 9:90.
239. Garrity T, Pandit R, Wright MA, Benefield J, Keni S, Young MR. Increased presence of CD34(+) cells in the peripheral blood of head and neck cancer patients and their differentiation into dendritic cells. Int J Cancer. 1997; 73:663–669.
240. Katou F, Ohtani H, Watanabe Y, Nakayama T, Yoshie O, Hashimoto K. Differing Phenotypes between Intraepithelial and Stromal Lymphocytes in Early-Stage Tongue Cancer. Cancer Res. 2007; 67:11195–11201.
241. Held W, Kijima M, Angelov G, Bessoles S. The function of natural killer cells: education, reminders and some good memories. Curr Opin Immunol. 2011; 23:228–233.
242. Cruz I, Meijer CJ, Walboomers JM, Snijders PJ, Van der Waal I. Lack of MHC class I surface expression on neoplastic cells and poor activation of the secretory pathway of cytotoxic cells in oral squamous cell carcinomas. Br J Cancer. 1999; 81:881–889.
243. Zancope E, Costa NL, Junqueira-Kipnis AP, Valadares MC, Silva TA, Leles CR, Mendonça EF, Batista AC. Differential infiltration of CD8+and NK cells in lip and oral cavity squamous cell carcinoma. J Oral Pathol Med. 2010; 39:162–167.
244. Bonecchi R, Bianchi G, Bordignon PP, D'Ambrosio D, Lang R, Borsatti A, Sozzani S, Allavena P, Gray PA, Mantovani A, Sinigaglia F. Differential expression of chemokine receptors and chemotactic responsiveness of type 1 T helper cells (Th1s) and Th2s. J Exp Med. 1998; 187:129–134.
245. Gaur P, Singh AK, Shukla NK, Das SN. Inter-relation of Th1, Th2, Th17 and Treg cytokines in oral cancer patients and their clinical significance. Hum Immunol. 2014; 75:330–337.
246. Agarwal A, Rani M, Saha GK, Valarmathi TM, Bahadur S, Mohanti BK, Das SN. Disregulated Expression of the Th2 Cytokine Gene in Patients with Intraoral Squamous Cell Carcinoma. Immunol Invest. 2003; 32:17–30.
247. Manchanda P, Sharma SC, Das SN. Differential regulation of IL-2 and IL-4 in patients with tobacco-related oral squamous cell carcinoma. Oral Dis. 2006; 12:455–462.
248. Sakaguchi S. Naturally arising CD4+ regulatory t cells for immunologic self-tolerance and negative control of immune responses. Annu Rev Immunol. 2004; 22:531–562.
249. Gasteiger G, Hemmers S, Firth MA, Le Floc'h A, Huse M, Sun JC, Rudensky AY. IL-2-dependent tuning of NK cell sensitivity for target cells is controlled by regulatory T cells. J Exp Med. 2013; 210:1167–1178.
250. Liu S, Liu D, Li J, Zhang D, Chen Q. Regulatory T cells in oral squamous cell carcinoma. J Oral Pathol Med. 2016; 45:635–639.
251. Schipmann S, Wermker K, Schulze HJ, Kleinheinz J, Brunner G. Cutaneous and oral squamous cell carcinoma-dual immunosuppression via recruitment of FOXP3+ regulatory T cells and endogenous tumour FOXP3 expression? J Craniomaxillofac Surg. 2014; 42:1827–1833.
252. Ming GL, Song H. Adult neurogenesis in the Mammalian brain: significant answers and significant questions. Neuron. 2011; 70:687–702.
253. Quan H, Fang L, Pan H, Deng Z, Gao S, Liu O, Wang Y, Hu Y, Fang X, Yao Z, Guo F, Lu R, Xia K, et al. An adaptive immune response driven by mature, antigen-experienced T and B cells within the microenvironment of oral squamous cell carcinoma. Int J Cancer. 2016; 138:2952–2962.
254. Takemori T. B Cell Memory, Plasma Cell Development. Molecular Biology of B Cells. 2015:227–249.
255. Loning T, Burkhardt A. Plasma cells and immunoglobulin-synthesis in oral precancer and cancer. Correlation with dysplasia, cancer differentiation, radio- and chemotherapy. Virchows Arch A Pathol Anat Histol. 1979; 384:109–120.
256. Halliley JL, Tipton CM, Liesveld J, Rosenberg AF, Darce J, Gregoretti IV, Popova L, Kaminiski D, Fucile CF, Albizua I, Kyu S, Chiang KY, Bradley KT, et al. Long-Lived Plasma Cells Are Contained within the CD19(-)CD38(hi)CD138(+) Subset in Human Bone Marrow. Immunity. 2015; 43:132–145.
257. von Allmen CE, Bauer M, Dietmeier K, Buser RB, Gwerder M, Muntwiler S, Utzinger S, Saudan P, Bachmann MF, Beerli RR. Identification of Ly-6K as a novel marker for mouse plasma cells. Mol Immunol. 2008; 45:2727–2733.
