# Supplementary material for: Microenvironmental regulation of the progression of oral potentially malignant disorders towards malignancy
Source: Oncotarget. 2017 Aug 17;8(46):81617–35. doi: 10.18632/oncotarget.20312 (PMC5655314; doi:10.18632/oncotarget.20312)
Supplement: Supplementary file 2 [file oncotarget-08-81617-s002.docx]

**Supplementary Table 1: Immune cell populations in the OPMD (using OLK for as an example) and OSCC microenvironment have distinct functions during the progression from OPMD to OSCC.**

| Cell  type | Markers  (human) | Markers  (mouse) | Function | Ref. | |
| --- | --- | --- | --- | --- | --- |
| Myeloid lineage | | | | | |
| MΦ | Pan-MΦ:  CD68  M1 MΦ:  CD80  M2 MΦ:  CD163 | CD11b^+^GR1^-^  CD68^+^CSF1R^+^  F4/80^+^ | Normal tissue: macrophages are able to phagocytize foreign objects, kill if necessary, and repair cellular damage. The classically ‘Inhibit’ type macrophages (called M1) are the primary host defense by killing pathogens, which means that they are pro-inflammatory. The alternatively activated ‘Heal’ type macrophages (M2) routinely repair and maintain tissue integrity. Thus, they are anti-inflammatory.  OLK tissue: possesses an M1 phenotype  OSCC tissue: possesses an M2 phenotype. M1 macrophages are anti-tumorigenic by producing pro-inflammatory cytokines and reactive intermediates such as oxygen and nitrogen (NO); M2 macrophages are pro-tumorigenic by inducing the expression of multiple receptors (mannose receptors and scavenging receptors), angiogenic factors such as VEGF, and low levels of pro-inflammatory cytokines. | [50, 51, 64, 66, 67, 220, 221] | |
| DC | CD11c^+^  CD83^+^  CD123^+^ | CD11c^+^  CD83^+^  CD123^+^ | Normal tissue: DCs are marrow-cells that are poised to sample the microenvironment and to transmit the gathered information to T cells and B cells.  OLK tissue: Dendritic Langerhans cells (LCs) is a subset of DCs present in oral mucosal linings. They can provide immunosurveillance to tissue compartments.  OSCC tissue: DCs engulf, process, and present TAAs to T-cells to generate tumor-specific cytotoxic T-cells. | [58, 93, 94, 222] | |
| TEM | CD11b^+^  SCA1^+^  TIE2^+^  CD14^+^  CD16^+^ | CD11b^+^  TIE2^+^  GR1^-^SCA1^+^ | Normal tissue: TIE2, a receptor for the angiogenic growth factor, is found almost exclusively on endothelial cells. It is required for normal embryonic vascular development.  OLK tissue: lack of evidence  OSCC tissue: TIE2-expressing monocytes (TEM) are increased in OSCC. They may play a role in tumor angiogenesis. | [68, 223, 224] | |
| Neutrophil | CD11b^+^CD66b^+^;  CD63^+^ | CD11b^+^  GR1^+^  7/4^+^ | Normal tissue: Neutrophils constitute the highest proportion of circulating leukocytes in humans and are phenotypically plastic.  OLK tissue: lack of evidence  OSCC tissue: Neutrophils are not expressed in normal oral mucosa or in OSCC, indicating that they may not function within the microenvironment. | [225, 226] | |
| Mast cell | CD11b^-^  CD49d^+^  CD117^-^  CD203c^+^ | CD11b^-^  CD49d^+^  CD117^-^  CD203c^+^ | Normal tissue: Mast cells play an important role during allergies and autoimmunity.  OLK tissue: Mast cells are recruited to pre-malignant lesions. The “Piece meal degranulation” of Mast cells aggravates the progression from OLK to OSCC.  OSCC tissue: Mast cells accumulate in OSCC and promote tumorigenesis by inducing angiogenesis, immunosuppression, releasing growth factors, and degradation of the extracellular matrix. | [162, 227] | |
| MDSC | CD11b^+^  CD33^+^  HLA-DR^-^  G-MDSC  ^Granulocytic^  CD14^-^CD15^+^  M-MDSC  ^monocytic^  CD14^+^ | CD11b^+^  GR1^+^  G-MDSC：^granulocytic^  Ly-6G^+^  Ly-6C^-^  CD49d^-^  M-MDSC:  ^monocytic^  Ly-6G^-^  Ly-6C^+^  CD49d^+^ | Normal tissue: MDSCs are absent in healthy hosts, but may naturally accumulate in situation of trauma and sepsis to temper immune responses.  OLK tissue: lack of evidence  OSCC tissue: The number of MDSCs increases in almost all cancer patients and animal models. MDSCs are immunosuppressive precursors of DCs, macrophages, and granulocytes. They can disrupt tumor immunosurveillance by disturbing the activation of T cells, their cytotoxic activity, and presentation of antigen and polarization of cells. | [68, 105, 228-239] | |
| Lymphoid lineage | | | | |  |
| NK cell | CD56^+^CD16^+^ | CD335^+^NK1.1^+^ | Normal tissue: NK cells are cytotoxic lymphocytes. When there is no antigen presentation, they can kill stressed cells.  OLK tissue: NK cells are present in low numbers.  OSCC tissue: NK cells are phenotypically inactivated related to those present in OSCC and their numbers are decreased. | [240-243] | |
| T_H_ cell | CD4  Th1: CCR5 CXCR3  Th2: CCR4 | CD3^+^CD4^+^ | Normal tissue: CD4^+^ T_H_ cells can be divided into T_H_1 and T_H_2 lineages.  OLK tissue: T_H_1-dominated microenvironment  OSCC tissue: T_H_1 cells secrete proinflammatory cytokines with the anti-tumorigenic functions of anti-tumorigenic. T_H_2 cells secrete anti-inflammatory cytokines with pro-tumorigenic functions. The ratio of T_H_1 to T_H_2 cells in OSCC correlates with cancer stage and grade; OSCC presents a T_H_1-dominated microenvironment. | [64, 244-247] | |
| T_reg_ cell | CD4^+^CD25^+^FOXP3^+^CTLA-4^+^CD45RA^+^ | CD4^+^CD25^+^FOXP3^+^CTLA-4^+^CD103^+^ | Normal tissue: T_regs_ play a critical role in the negative regulation of adaptive immune system by expansion and activation of T and B cells, mediation of immune homeostasis and self-tolerance.  OLK tissue: lack of evidence  OSCC tissue: The function of T_regs_ in oral carcinoma is controversial. | [248-251] | |
| T_c_ cell | CD3^+^CD8^+^ | CD3^+^CD8^+^ | Normal tissue: CD8^+^ cytotoxic T (T_C_) cells are the main effector cells of adaptive immunity.  OLK tissue: Proliferation of dysplastic cells and activation of immune system are in an equilibrium phase.  OSCC tissue: CD8^+^ cells can recognize tumor antigens and destroy cancer cells. | [99, 243] | |
| B cell | CD19^+^CD20^+^ | B220^+^CD19^+^CD22^+^ | Normal tissue: B cells are essential parts for humoral immunity.  OLK tissue: B-lymphocytes are significantly prominent in severe dysplasia.  OSCC tissue: They can promote OSCC by secreting pro-tumorigenic cytokines and altering T_H_1 to T_H_2 ratios. | [252, 253] | |
| Plasma cell | CD19^-^CD38^hi^CD138^+^ | mLy-6K | Normal tissue: Plasma cells are specialized forms of antibody-producing B cells, which can synthesize and secrete immunoglobulins.  OLK tissue: Immunoglobulin-labeled plasma cells (IgA and IgG) are localized throughout all epithelial layers in OLK with dysplasia.  OSCC tissue: The number of plasma cells, especially IgA- and IgG-containing plasma cells, decreases significantly with progressive tumor dedifferentiation. | [254-257] | |

MΦ: macrophages; DC: dendritic cells; TEM: TIE2-expressing monocytes; VEGF: vascular endothelial growth factor; TAAs: tumor-associated antigens; MDSC: myeloid-derived suppressor cells; NK cell: natural killer cell
